# The Effects of a Cinchona Supplementation on Satiety, Weight Loss and Body Composition in a Population of Overweight/Obese Adults: A Controlled Randomized Study

**DOI:** 10.3390/nu15245033

**Published:** 2023-12-07

**Authors:** Martina Chiurazzi, Barbara De Conno, Mariastella Di Lauro, Bruna Guida, Gilda Nasti, Elisabetta Schiano, Mariano Stornaiuolo, Gian Carlo Tenore, Antonio Colantuoni, Ettore Novellino

**Affiliations:** 1 Department of Clinical Medicine and Surgery, University of Naples “Federico II”, 80131 Naples, Italy; martina.chiurazzi88@gmail.com (M.C.); msdl@hotmail.it (M.D.L.); bguida@unina.it (B.G.); gildanasti@libero.it (G.N.); anto.colanti@gmail.com (A.C.); 2 Department of Medical Oncology, AO “A. Cardarelli”, 80131 Naples, Italy; 3 Department of Pharmacy, University of Napoli “Federico II”, 80131 Naples, Italy; mariano.stornaiuolo@unina.it (M.S.); gctenore@unina.it (G.C.T.); 4 Inventia Biotech Centro Ricerche Alimentari Healthcare, 81120 Caserta, Italy; elisabettaschiano@inventiabiotech.com (E.S.); ettore.novellino@unicatt.it (E.N.); 5 Department of Medicine and Surgery, Catholic University of the Sacred Heart, 00168 Rome, Italy

**Keywords:** obesity, cinchona, nutraceutical treatment, body composition analysis, gut hormone, bitter taste

## Abstract

Obesity is a risk factor for several diseases present worldwide. Currently, dietary changes and physical activity are considered the most effective treatment to reduce obesity and its associated comorbidities. To promote weight loss, hypocaloric diets can be supported by nutraceuticals. The aim of this study was to evaluate the effects of a hypocaloric diet associated with *Cinchona succirubra* supplementation on satiety, body weight and body composition in obese subjects. Fifty-nine overweight/obese adults, were recruited, randomized into two groups and treated for 2 months. The first group (32 adults) was treated with a hypocaloric diet plus cinchona supplementation (the T-group); the second one (27 adults) was treated with a hypocaloric diet plus a placebo supplementation (the P-group). Anthropometric-measurements as well as bioimpedance analysis, a Zung test and biochemical parameters were evaluated at baseline and after 60 days. T-group adults showed significant improvement in nutritional status and body composition compared to those at the baseline and in the P-group. Moreover, T-group adults did not show a reduction in Cholecystokinin serum levels compared to those of P-group adults. In conclusion, our data demonstrate that a hypocaloric diet associated with cinchona supplementation is effective in inducing more significant weight loss and the re-establishment of metabolic parameters than those obtained with a hypocaloric diet.

## 1. Introduction

Obesity is a chronic disease present worldwide, known to increase the risk factors for many pathological conditions, such as cardiovascular diseases, hypertension, infertility, diabetes mellitus and dyslipidemia [1,2]. The World Health Organization (WHO) has defined obesity as an abnormal or excessive accumulation of fat that represents a health risk, as indicated by a body mass index (BMI) ≥ 30 kg/m^2^, while a body mass index (BMI) over 25 kg/m^2^ is considered overweight [3]. Previous studies have shown that a weight loss of 5–10% at baseline in subjects with obesity associated with other comorbidities can significantly improve health and counteract mortality [4]. The etiology of obesity is multifactorial, involving, indeed, genetic and hormonal factors as well as social and environmental factors, mainly represented by a sedentary lifestyle and unhealthy eating habits [4,5,6].

Gut hormones play an important role in the regulation of energy balance; they, indeed, modulate feeding behavior as well as energy expenditure and nutrient partitioning [7]. Recently, it has been shown that gut hormone secretion can be regulated by G-protein-coupled taste receptor (TAS2R) family 2, a family of taste receptors activated by bitter molecules. Evolutionally, TAS2R has played important roles allowing organisms to detect potentially harmful substances and to avoid their ingestion. Being allocated in the gastrointestinal tract, it plays important roles in intestinal chemoreception [8], coupling bitter molecules binding to gut hormone secretion.

The expression of TAS2Rs varies among people and there exist interindividual variations associated with gut hormone secretion upon taste receptor stimulation [9,10]. Previous in vivo studies indicate that the stimulation of TAS2R family members expressed by enteroendocrine cells (ECCs) is effective in inducing the release of gastrointestinal peptides, such as CCK and GLP-1, which are involved in the control of the sense of hunger and satiety, and sequentially of food intake.

The CCK hormone, released by the duodenal I cell after the consumption of a meal rich in lipids, is the most studied peptide up to date that is able to inhibit the sense of hunger and mediated by CCK1 receptors on vagal afferents [11,12].

Food intake and/or the ingestion of bitter substances can result in an increase in the release of CCK and a reduction in hormones, such as ghrelin and motilin, causing a decrease in gastrointestinal motility, in the hunger sense and, finally, in food intake [13]. The effects on the secretion of gastrointestinal (GI) hormones and consequently on the slowing of gastric emptying play an important role in regulating energy intake, representing a new approach to the management or prevention of obesity and its comorbidity [14,15,16].

To date, the common interest is to find a treatment that can counteract or prevent obesity, promoting and protecting individual health. A large amount of data indicate that a healthy diet and an active lifestyle play a key role in the prevention and treatment of obesity [17,18]. In particular, several dietary supplements, commonly called nutraceuticals, have been shown to have health benefits [19,20]. Furthermore, nutraceutical use associated with an appropriate diet and daily physical activity can represent an effective treatment for metabolic syndrome [21,22]. The aim of the present study was to assess the effects of a nutraceutical supplementation with *Cinchona succirubra* (cinchona), a bitter taste agonist, on a population of overweight/obese adults. Cinchona is an arboreal plant belonging to the Rubiaceae family, from South America; in the past, this natural compound was widely used as a pharmacological treatment for its anti-malarial, analgesic and anti-flu properties [23,24,25,26,27]. Previous studies demonstrate the beneficial effects of dietary cinchonine in preventing obesity [25]; in particular, our purpose was to evaluate nutraceutical effects on satiety, weight loss, changes in body composition and nutritional status in a group of overweight/obese adults.

## 2. Materials and Methods

### 2.1. Study Design

The study protocol was approved by the Ethical Committee of the Federico II University Medical School of Naples—A.O.R.N. Cardarelli (EC approval code: 204/2023); all adults gave written informed consent. The clinical trial was registered at www.isrctn.com, accessed on 25 July 2023 (ISRCTN 13055163 number).

Overweight/obese adults attending Outpatients Clinic of the Departmental program “Diet therapy in transplantation and chronic renal failure”, School of Medicine, “Federico II” University of Naples, were recruited. A low-calorie diet was recommended for all overweight/obese adults.

### 2.2. Inclusion and Exclusion Criteria

The adults eligible for the study were over 18 years of age with overweight/obesity, attending Outpatients Clinic of the Departmental program “Diet therapy in transplantation and chronic renal failure”, School of Medicine, “Federico II” University of Naples. Overweight/obesity was defined by a BMI > 25 and <45 kg/m^2^.

Adults were ineligible if the following were observed:

- They participated in other clinical trials;
- They declined to consent;
- They showed a weight change of more than 3 kg in the last 2 months;
- They were treated with weight loss medications and/or had a history of bariatric surgery;
- They were treated with hormonal therapies (estrogens, thyroxine or progesterone);
- They currently have cancer or received a cancer diagnosis within the last 5 years;
- They suffered with acute or chronic metabolic and inflammatory diseases (Crohn’s disease, rheumatoid arthritis, etc.);
- They suffered from type 1 or type 2 diabetes or had been treated with insulin and oral hypoglycemic drugs (changes in the regulation of intermediary metabolism).

Patients with type 2 diabetes, treated with diet alone, could be included in the study.

After baseline evaluations, subjects were randomized into 2 groups: the first group (32 adults) was treated with a hypocaloric diet for 2 months plus the supplementation of cinchona (T group); the second one (27 adults) was treated with a hypocaloric diet for 2 months plus a placebo supplementation (P-group). Adults affected by diseases, such as those reported above, cancer, acute and chronic metabolic and inflammatory diseases, type 1 and type 2 diabetes treated with insulin and/or hypoglycemic drugs or treated with drugs to lose weight or treated with hormone therapy were excluded.

### 2.3. Dietary Treatment

A personalized diet was recommended for each patient of both groups, om accordance with LARN (Livelli di Assunzione Raccomandata di Nutrienti) guidelines [28,29]. A hypocaloric diet (calorie restriction was 40% of the total energy needs), with 55–60% of the total caloric intake comprising carbohydrates, 10–15% of that comprising proteins and 20–25% of that being composed of fatty acids (<7% from saturated fat), was recommended for all obese adults.

### 2.4. Supplementation

The consumption of cinchona is not associated with health risks [30,31,32].

The *Cinchona succirubra* bark powder used for the nutraceutical formulation was provided by NGN Healthcare New Generation Nutraceuticals srl. To allow the nutraceuticals to exclusively address duodenal EECs, the nutraceuticals were encapsulated in gastro-resistant capsules containing 400 mg of *Cinchona succirubra* bark powder microencapsulated with maltodextrins (two cinchona capsules per day, one hour before main meals). The placebo capsules used were produced using only maltodextrins and administered in accordance with the same methods. The subjects belonging to the T-group were treated with 800 mg of cinchona per day (two cinchona capsules per day, one hour before main meals). Participants who did not take pills for two or more days were excluded from the study.

### 2.5. Study Protocol

The subjects were evaluated at the moment of recruitment (time T0), after 30 (time T1) and 60 (time T2) days of treatment, using standardized protocols.

Nutritional status was assessed via anthropometric measurements: weight (Seca GmbH & Co. KG, Hamburg, Germany), height (measured using a wall-mounted stadiometer to the nearest 0.1 cm), body mass index (BMI), waist circumference (WC) and hip circumference (HC) [33]. In particular, total body water (TBW), fat mass (FM) and fat-free mass (FFM) percentages were detected to evaluate the body composition, via bioelectrical impedance analysis (BIA) undertaken using a tetrapolar BIA (RJL 101; Akern SRL, Florence, Italy). BIA was performed with a single-frequency measurement (50 kHz) [34]. The Zung test for assessing well-being was performed at baseline and at the end of the study. The Zung test represents a 20-item Likert scale, with scores ranging from 20, ‘‘no depression’’, to 80, ‘‘major depression’’, which are converted into index scores by dividing the sum of the raw scores by 80 and multiplying the result by 100 [35].

**Figure 1:**
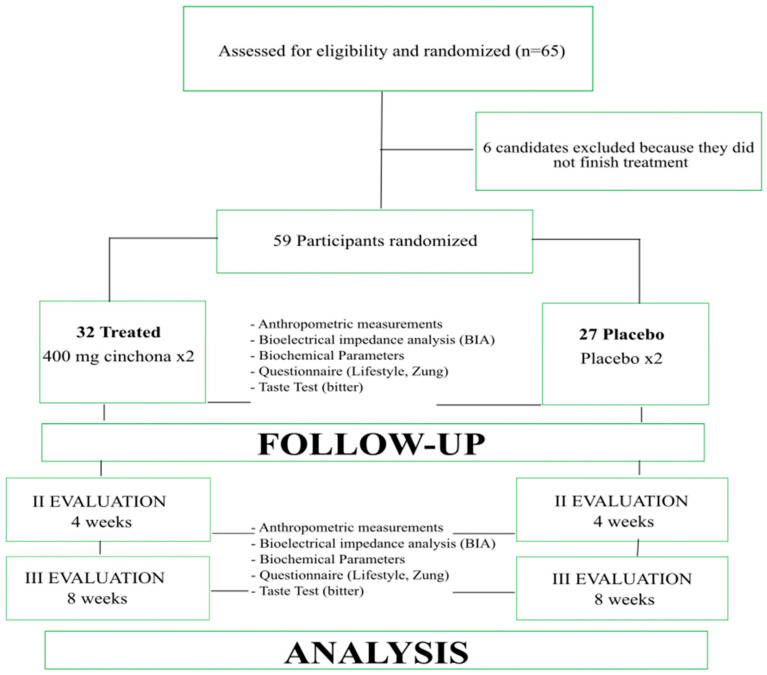
Flow chart illustrating study population.

Blood glucose (Gly), total cholesterol (TC), HDL cholesterol (HDL-c), LDL cholesterol (LDL-c), triglycerides (TG), uric acid, alanine amino transferase (ALT) and aspartate aminotransferase (AST) were measured and monitored during the treatment. All subjects, at T0 and at the end of the treatment, were tested for bitter taste to classify the subjects as tasters or non-tasters [36] (Figure 1).

### 2.6. Cinchona Alkaloid Status Assessment

To determine cinchona tasting status, all subjects consecutively placed five paper strips on their tongue; the first one was a control strip, whereas the others were impregnated with 0.4, 0.9, 2.4 and 6.0 mg of quinine extract, respectively. “Tasters” were defined as subjects who perceived bitter taste from any of the quinine impregnated blotting paper strips.

### 2.7. Dot Blot Protocol for CCK and Ghrelin Analyses

Serum CCK and ghrelin levels were measured via Dot-blot. Briefly, five microliters of serum samples was spotted on a nitrocellulose membrane to then be processed via Western blotting. CCK and ghrelin levels were immunodetected using anti-ghrelin (Santa Cruz, CA, USA, dilution 1:250) and anti-cholecystokinin (Santa Cruz, CA, USA, dilution 1:250) anti-bodies.

### 2.8. Compliance

Compliance with the dietary intervention was assessed by monitoring the dietary intake at baseline and every month until the end of the study using food frequency questionnaires [37].

The assessment of compliance with physical activity was verified by asking subjects to complete a physical activity questionnaire [38]. The questionnaires confirmed that adults belonging to both groups carried out regular physical activity for the entire duration of the treatment.

Satiety was assessed by means of visual analog scales (VAS), a previously validated questionnaire [39].

Compliance with the nutraceutical supplementations was evaluated using a daily questionnaire asking each volunteer about the time of the consumption of the supplement as well as evaluating the presence of adverse events.

In addition, the number of capsules at the end of the study was recorded.

### 2.9. Sample Size and Statistical Power

Sample size calculation was performed using MedCalc. The outcome for the calculation of sample size was the reduction in body weight in the T-group. A difference of 25% between the T-group and P-group was estimated. Power and significance levels were set at 0.80 and 0.05, respectively. Using these parameters, the estimated sample size was 30 participants per group.

### 2.10. Statistical Analysis

All data were expressed as the mean ± standard error of the mean (SEM). Data were analyzed with the IBM SPSS 20.0 program (Statistical Package for Social Science, SPSS, Chicago, IL, USA). A paired-sample *t*-test, independent-sample *t*-test and chi-squared test were performed. Hormone levels were compared among groups using ordinary two-way ANOVA, correcting for multiple comparisons, and controlling the false discovery rate using the original FDR method of Benjamini and Hochberg [40,41]. Statistical significance was set at *p* < 0.05.

## 3. Results

### 3.1. Nutritional Status Evaluation

**Figure 2:**
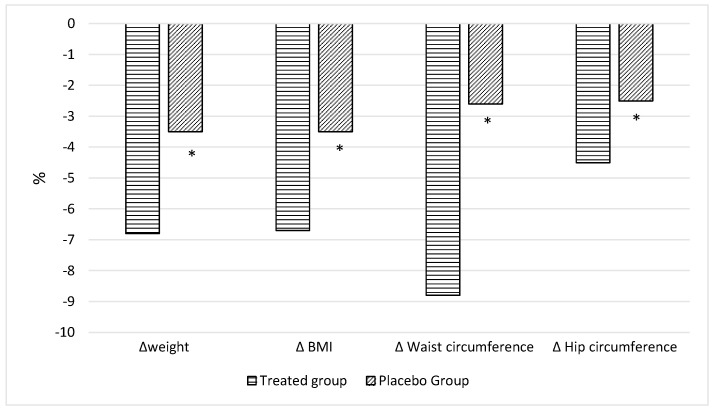
Percentage variations in anthropometric measurements in treated and placebo group compared with baseline. Data are reported as mean. * *p* < 0.05; T0, placebo group vs. treated group.

**Figure 3:**
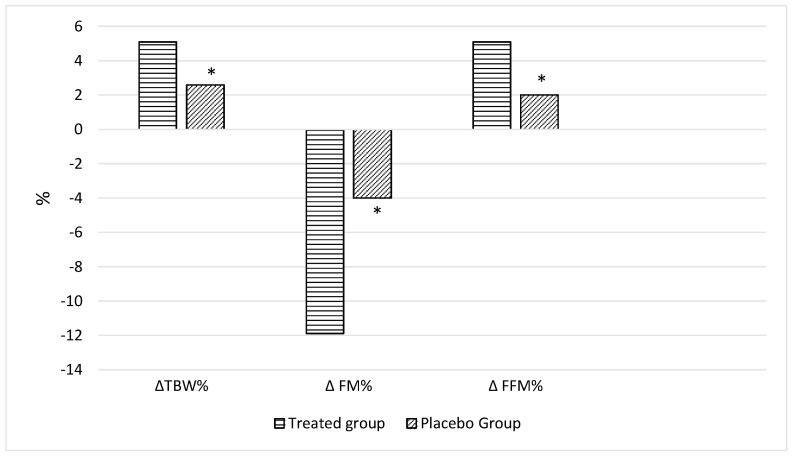
Total body water (TBW), fat mass (FM) and fat-free mass (FFM) percentage variations in treated and placebo group compared with those at baseline. Data are reported as mean. * *p* < 0.05; placebo group vs. treated group.

All adults were accurately evaluated at baseline and were reconsidered after 30 and 60 days. No significant differences in all parameters were observed at the baseline among the two groups with the exception of hip circumference (P-group vs. T-group: *p* = 0.019) and the percentage of total body water (P-group vs. T-group: *p* = 0.49) (Table 1). On the basis of the dietary questionnaire, in the whole cohort, all adults had high adherence to the Med-Diet. At the end of the observations, the adults belonging to T-group presented significant changes in nutritional status and body composition compared to those at T0 (Table 2) and a higher improvement compared to those in the P-groups (Table 2), in particular in body weight (*p* < 0.0001 Table 2, Figure 2) as well as in BMI (*p* < 0.0001, Table 2, Figure 2), waist circumference (*p* = 0.047, Table 2, Figure 2), hip circumference (*p* = 0.016, Table 2, Figure 2), FM% (*p* = 0.013, Table 2, Figure 3), FFM% (*p* = 0.006, Table 2, Figure 3) and TBW% (*p* = 0.004, Table 2, Figure 3). Zung scores for adults belonging to both groups were less than 45 and therefore did not indicate a depression, at the end of the treatment. However, a significant reduction was observed in both the treated (*p* < 0.0001) and the placebo group (*p* = 0.008) (Table 2). Biochemical parameters were monitored throughout the study; parameters, such as HDL-c, and both transaminases remained stable throughout the study in both groups. Moreover, adults perceived the bitter taste of quinine-impregnated blotting paper and were thus all classified as “Tasters in both groups”.

Furthermore, satiety questionnaires administered in both groups revealed that the T-group showed greater satiety throughout the duration of treatment compared with that in the P-group confirming the data obtained via hormonal measurements.

VAS analysis revealed that, compared to those in the P-group, adults belonging to the T-group felt more satiated before (3.5 + −0.9 and 4.2 + −0.6 for the P- and T-group, respectively; *p* value = 0.0059) and during the meals (3.3 + −0.7 and 4.3 + −0.7 for the P- and T-group, respectively; *p* value < 0.0001), suggesting that cinchona prolonged satiety throughout the day.

### 3.2. Treated Group (T-Group)

Nutraceutical supplementation improved the nutritional status of adults belonging to the T-group, inducing a significant reduction in Body weight (90.4 ± 1.7 vs. 96.9 ± 1.6 kg, *p* < 0.0001; T2 vs. T0) as well as in BMI (32.4 ± 1.1 vs. 34.6 ± 1.0 kg/m^2^, *p* < 0.0001; T2 vs. T0), waist circumference (96.1 ± 1.0 vs. 102.7 ± 1.1 cm, *p* < 0.0001; T2 vs. T0) and hip circumference (115.2 ± 1.1 vs. 120.7 ± 1.1 cm, *p* < 0.0001; T2 vs. T0) when compared with those at baseline. Finally, bioimpedance analysis showed a significant reduction in FM% (31.1 ± 2.1% vs. 34.6 ± 1.7%; *p* < 0.0001, T2 vs. T0) in the T-group after 2 months of nutraceutical supplementation. There was, moreover, a significant increase in FFM% (68.9 ± 1.4% vs. 65.4 ± 1.2%, *p* < 0.0001; T2 vs. T0) and TBW% (50.6 ± 1.2% vs. 48.0 ± 1.1%, *p* < 0.0001; T2 vs. T0) when compared with those at baseline.

It is interesting to observe the bioumoral parameter improvements; in particular, significant reductions in glucose (87.4 ± 1.0 vs. 92.6 ± 1.4 mg/dL, *p* = 0.025; T2 vs. T0), TC (173.4 ± 2.9 vs. 189.2 ± 3.0 mg/dL, *p* = 0.04; T2 vs. T0), LDL-c (106.7 ± 3.5 vs. 118.9 ± 3.5 mg/dL, *p* = 0.029; T2 vs. T0) and uric acid (3.3 ± 0.7 vs. 3.9 ± 0.6 mg/dL, *p* = 0.07; T2 vs. T0) were detected when compared with those at baseline. Conversely, HDL-c as well as Try, both transaminases, PA, SM and SMI remained stable throughout the study (Table 2).

### 3.3. Placebo Group (P-Group)

Adults belonging to the P-group showed an improvement in body weight (87.5 ± 2.0 vs. 90.7 ± 2.0 kg, *p* < 0.0001; T2 vs. T0) as well as in BMI (31.8 ± 0.8 vs. 33.0 ± 0.8 kg/m^2^, *p* < 0.0001; T2 vs. T0). Furthermore, a significant reduction in waist circumference (98.9 ± 1.4 vs. 100.7 ± 1.5 cm, *p* < 0.0001; T2 vs. T0) as well as in hip circumference (111.7 ± 0.7 vs. 113.7 ± 0.9 cm, *p* = 0.001; T2 vs. T0) was detected after 60 days of treatment. Finally, bioimpedance analysis showed a significant reduction in FM% (28.4 ± 1.6 vs. 29.8 ± 1.6%; *p* = 0.02, T2 vs. T0) in the P-group after 60 days of placebo supplementation and an increase in FFM% (71.5 ± 1.0 vs. 70.2 ± 1.0%, *p* = 0.01; T2 vs. T0). Conversely, no significant differences were detected in TBW%, PA, SM and SMI compared with those at baseline. A significant improvement was detected in bioumoral parameters after 60 days; in particular, significant reductions in glucose (91.1 ± 1.3 vs. 96.0 ± 1.6 mg/dL, *p* = 0.001; T2 vs. T0), TC (171.1 ± 2.8 vs. 185.9 ± 3.2 mg/dL, *p* = 0.05 T2; vs. T0), LDL-c (106.7 ± 3.0 vs. 122.3 ± 3.5 mg/dL, *p* = 0.035 T2; vs. T0), Try (84.6 ± 3.8 vs. 109.5 ± 6.8 mg/dL, *p* = 0.023; T2 vs. T0) and uric acid (3.0 ± 0.6 vs. 4.1 ± 0.8 mg/dL, *p* = 0.010; T2 vs. T0) were observed compared with those at baseline. Conversely, HDL-c and both transaminases remained stable throughout the study (Table 2).

### 3.4. Serum CCK and Ghrelin Level

To confirm nutraceutical supplementation components addressing enteroendocrine cells (EECs), CCK and ghrelin serum levels were measured in treated and placebo groups, both at T0 and T2. Previously, we showed that bitter taste receptors are expressed in gastric EECs as well as in intestinal EECs. Gastric EECs secrete ghrelin while intestinal EECs secrete CCK [42,43]. Considering that nutraceutical supplementation was administered with gastro-resistant pills, we expected the stimulation of intestinal EECs but not of gastric EECs. As shown in Table 3 at T0 and T2, placebo and treated adult serum samples were processed via Dot-blot to detect circulating CCK and ghrelin. It is worth noting that the hypocaloric diet decreased CCK levels at T2 in placebo adults (delta −40% ± 10, *p* = 0.046); this reduction has been linked to increased hunger in adults undergoing hypocaloric diets. On the contrary, in adults receiving the nutraceutical supplementation, we did not measure a statistically significant reduction in CCK levels, indicating that nutraceuticals were effective in stimulating intestinal EECs to release CCK. Moreover, we measured ghrelin levels, which were unchanged in both placebo and treated group at the end of observations, indicating that there was no stimulation of gastric EECs.

## 4. Discussion

The results of the present study indicate that overweight/obese adults attending Outpatients Clinic of the Departmental program “Diet therapy in transplantation and chronic renal failure”, School of Medicine, “Federico II” University of Naples, treated with the supplementation of *Cinchona succirubra*, showed a significant improvement in nutritional status and body composition after 60 days of treatment compared with baseline. These adults were compared with a group of overweight/obese adults treated with a hypocaloric diet without supplementation. Interestingly, the cinchona-treated adults showed a reduction in body weight, BMI, waist circumference, hip circumference and FM% that was higher than that detected in the subjects receiving the placebo. Furthermore, a higher improvement in the percentage of FFM and TBW was observed in the T-group, when compared with baseline and adults belonging to the P-group. This study, indeed, demonstrates that the association of hypocaloric diet with cinchona nutraceutical supplementation is effective in inducing higher weight loss than that obtained with a hypocaloric diet.

The study justified the choice of *Cinchona succirubra* as a dietary supplement in the broader context of the use of natural substances or nutraceuticals for the treatment of obesity and weight control. The selection of cinchona was influenced by its role as a bitter taste agonist. This property was considered potentially beneficial for modulating food intake, therefore leading to improved weight management. In this context, previous preclinical studies strongly supported our selection.

Our results are in agreement with those of a previous study where Jung and coworkers investigated the effects of dietary cinchonine on the reduction in high-fat-diet- (HFD) induced adipogenesis and inflammation in a mouse model. In particular, the authors showed that HFD-fed mice treated with 0.05% dietary cinchonine for 10 weeks presented a reduction in body weight as well as an improvement in blood parameters, such as triglycerides, cholesterol and glycemia, accompanied by an attenuation in proinflammatory cytokine production. Therefore, the authors suggested that cinchona is able to prevent obesity due to its effects on adipogenesis and inflammation [25].

Furthermore, adults belonging to the treated group showed a surprising reduction in the percentage of FM compared with that in the placebo group, confirming previous data presented by Cettour-Rose and coworkers, who studied the effect of 0.1% quinine on body weight and body composition in male mice fed a balanced diet. In particular, they showed that quinine, a cinchona alkaloid belonging to the aryl-amino alcohol group of drugs, is effective in the management and control of body weight and fat mass without affecting food intake. Therefore, the authors indicated that this could represent a novel tool to counteract obesity [44]. Our present data support the previous suggestions, indicating that the stimulation of taste receptors by nutraceuticals based on cinchona affect the complex interplay of molecules involved in the regulation of food intake, body metabolism and total energy introduction and expenditure, facilitating real control of individual body weight.

It is interesting that adults belonging to the treated group revealed greater satiety throughout the duration of treatment, suggesting that nutraceutical treatment with cinchona is able to modulate gastrointestinal (GI) functions, including intestinal hormones, leading to an increased satiety and the consequent fine modulation of energy intake.

The results of this study indicate that the treatment with cinchona plus hypocaloric diet was accompanied by an interesting resetting of hormonal secretion by intestinal endocrine cells. The hypocaloric diet in the P-group, indeed, induced a decrease in CCK secretion and blood levels, as previously observed in the induction of weight loss via hypocaloric diet treatment. However, it is worth noting that the chinchona supplementation plus the hypocaloric diet did not modify the CCK secretion and blood levels, suggesting that cinchona was effective in facilitating CCK secretion, which was sequentially accompanied by higher satiety in the adults, as detected in our T-group. Therefore, CCK secretion and unmodified blood levels trigger the adaptation to weight loss induced by cinchona, as characterized by a decrease in hunger stimulation and better compliance to the hypocaloric diet. This likely suggests that this adaptation facilitates weight loss with a real improvement in all the morphometric and functional parameters under all time periods of treatment in our treated cases compared to those in the placebo group. On the other hand, in the P-group, the decrease in CCK secretion and blood levels indicate that the risk of hunger stimulation may play a key role in major alterations or failure in weight loss management.

In conclusion, weight loss management triggers the adaptation of circulating hormones, which is characterized by a decrease in CCK secretion with the consequent risk of failure of weight loss maintenance. Supplementation with cinchona in a hypocaloric diet facilitates higher circulating levels of CCK, which are effective in promoting patient satiety and reducing the risk of hunger stimulation and failure in weight loss attempts. These data call for further studies to clarify the complex interplay of different gut-derived hormones in the modulation of food and energy intake.

## 5. Limitation

Although this study presents promising results regarding the efficacy of *Cinchona succirubra* supplementation in conjunction with a hypocaloric diet for weight loss in overweight/obese patients, there are several limitations that must be acknowledged. First, the sample size and demographic diversity of the study were limited, calling for further research to confirm these findings across a broader population. Additionally, the study duration was relatively short (60 days), indicating the necessity for longer-term studies to evaluate the sustainability and prolonged effects of such treatment. While the study offers promising initial results for morphometric and functional parameters, an in-depth exploration of the long-term implications and potential side effects would be really useful. Furthermore, the biochemical mechanisms contributing to the observed weight loss and hormonal regulation also require further detailed investigation. Finally, while the study suggests an interesting resetting of hormonal secretion and appetite control, these results need to be verified with larger and diverse study populations over longer periods of time to validate the efficacy and safety of cinchona supplementation and the complex interplay of different gastro-intestinal hormones in weight management.

In this manuscript, we only measure fasting levels of CCK and ghrelin in adults receiving *Cinchona succirubra*. In the future, it will be interesting to measure postprandial levels of these appetite hormones upon *Cinchona succirubra* supplementation. Their levels will confirm the stimulatory mechanism of action of cinchona on intestinal bitter receptors and/or eventually highlight an unprecedented mechanism of action involving long-term transcriptional modulatory activity on enteroendocrine cells.

## 6. Conclusions

The results obtained from this study pave the way for a range of future research opportunities, which are crucial to deepening our understanding and applications in clinical settings. One immediate area to explore is the longitudinal evaluation of the effects of cinchona on weight and body metabolism. Studies extending over longer periods will be critical to assess the long-term safety and efficacy of this dietary supplement. The diversification of the study population in future trials is also essential. Including participants from various demographic backgrounds, different age groups and ethnicities will help determine the effectiveness of cinchona supplementation across a broader spectrum of the population. Furthermore, the mechanisms triggered by cinchona, affecting weight loss and metabolic parameters, need to be clarified. Such studies would provide valuable insights into its role in appetite regulation and energy balance, contributing to an understanding of its therapeutic potential. Significantly, comparative studies will also be interesting in terms of assessing the efficacy of cinchona, when combined with other plant matrices, as sources of alkaloids able to activate bitter taste receptors. Such combinations could potentially enhance the beneficial potential or mitigate any adverse effects, offering a new approach to weight management.

## Figures and Tables

**Table 1:** Baseline clinical characteristics of participants. Data are reported as mean ± SEM. * *p* < 0.05, T0 placebo group vs. T0 treated group. Abbreviations: total cholesterol (TC), high-density lipoprotein (HDL-c), low-density lipoprotein (LDL-c), triglycerides (TG), aspartate aminotransferase (AST), alanine aminotransferase (ALT), total body water (TBW), fat mass (FM), fat-free mass (FFM), phase angle (PA), skeletal mass (SM) and skeletal mass index (SMI).

	Nutritional Assessment (T0)	
	Treated Group (n = 32)	Placebo Group (n = 27)
**Male, n° (%)**	9 (28.1)	11 (44.7)
**Female, n° (%)**	23 (71.9)	16 (55.3)
**Age, years**	36.7 ± 2.1	40.4 ± 1.9
**Body Weight, kg**	96.9 ± 1.6	90.7 ± 2.0
**BMI, kg/m^2^**	34.6 ± 1.0	33.0 ± 0.8
**Waist circumference, cm**	102.7 ± 1.1	100.7 ± 1.5
**Hip circumference, cm**	120.7 ± 1.1	113.7 ± 0.9 *
**% Taste**	100%	100%
	**Comorbidities**	
	**Treated Group** **(n = 32)**	**Placebo Group** **(n = 27)**
**ZUNG**	39.2 ± 1.2	37.3 ± 1.3
**DIABETES n (%)**		
No prediabetes or diabetes	30 (93.8)	22 (81.5)
Prediabetes	2 (6.2)	4 (14.8)
Diabetes	0 (0)	1 (3.7)
	**Plasma Metabolic Parameters (T0)**	
	**Treated Group** **(n = 32)**	**Placebo Group** **(n = 27)**
**Glucose, mg/dL**	92.6 ± 1.4	96.0 ± 1.6
**TC, mg/dL**	189.2 ± 3.0	185.9 ± 3.2
**HDL-c, mg/dL**	46.5 ± 1.8	46.6 ± 1.4
**LDL-c, mg/dL**	118.9 ± 3.5	122.3 ± 3.5
**TG/HDL-c ratio**	2.5 ± 0.7	2.5 ± 1.3
**TG, mg/dL**	108.0 ± 3.7	109.5 ± 6.8
**AST**	22.3 ± 2.3	22.4 ± 1.5
**ALT**	24.5 ± 3.3	24.7 ± 2.3
**Uric Acid**	3.9 ± 0.6	4.1 ± 0.8
**TBW%**	48.0 ± 1.1	51.6 ± 0.8 *
**FM%**	34.6 ± 1.7	29.8 ± 1.6
**FFM%**	65.4 ± 1.2	70.2 ± 1.0
**PA**	7.0 ± 0.3	7.3 ± 0.5
**SM**	35.7 ± 1.1	36.5 ± 1.5
**SMI**	12.6 ± 0.4	13.3 ± 0.6

**Table 2:** Anthropometric measurements as well as biochemical parameters and body composition at baseline and after 2 months of treatment. Data are reported as mean ± SEM. * *p* < 0.05, T2 vs. T0; ° < 0.05, placebo group vs. treated group.

	Nutritional Assessment			
	Treated Group (n = 32)		Placebo Group (n = 27)	
	**T0**	**T2**	**T0**	**T2**
**Body Weight, kg**	96.9 ± 1.6	90.4 ± 1.7 *	90.7 ± 2.0	87.5 ± 2.0 *°
**BMI, kg/m^2^**	34.6 ± 1.0	32.4 ± 1.1 *	33.0 ± 0.8	31.8 ± 0.8 *°
**Waist circumference, cm**	102.7 ± 1.1	96.1 ± 1.0 *	100.7 ± 1.5	98.9 ± 1.4 *°
**Hip circumference, cm**	120.7 ± 1.1	115.2 ± 1.1 *	113.7 ± 0.9	111.7 ± 0.7 *°
**ZUNG**	39.2 ± 1.2	34.6 ± 1.3 *	37.3 ± 1.3	35 ± 1.4 *
	**Plasma Metabolic Parameteres**			
	**Treated Group** **(n = 32)**		**Placebo Group** **(n = 27)**	
	**T0**	**T2**	**T0**	**T2**
**Glucose, mg/dL**	92.6 ± 1.4	87.4 ± 1.0 *	96.0 ± 1.6	91.1 ± 1.3 *
**TC, mg/dL**	189.2 ± 3.0	173.4 ± 2.9 *	185.9 ± 3.2	171.1 ± 2.8 *
**HDL-c, mg/dL**	46.5 ± 1.8	45.3 ± 1.6	46.6 ± 1.4	47.5 ± 1.7
**LDL-c, mg/dL**	118.9 ± 3.5	106.7 ± 3.5 *	122.3 ± 3.5	106.7 ± 3.0 *
**TG/HDL ratio**	2.5 ± 0.7	2.4 ± 0.8	2.5 ± 1.3	2.0 ± 0.8
**TG, mg/dL**	108.0 ± 3.7	103.8 ± 4.5	109.5 ± 6.8	84.6 ± 3.8 *
**AST**	22.3 ± 2.3	21.4 ± 1.4	22.4 ± 1.5	21.1 ± 1.6
**ALT**	24.5 ± 3.3	21.6 ± 2.0	24.7 ± 2.3	22.5 ± 2.4
**Uric Acid**	3.9 ± 0.6	3.3 ± 0.7 *	4.1 ± 0.8	3.0 ± 0.6 *
**TBW%**	48.0 ± 1.1	50.6 ± 1.2 *	51.6 ± 0.8	52.4 ± 0.8 °
**FM%**	34.6 ± 1.7	31.1 ± 2.1 *	29.8 ± 1.6	28.4 ± 1.6 *°
**FFM%**	65.4 ± 1.2	68.9 ± 1.4 *	70.2 ± 1.0	71.5 ± 1.0 *°
**PA**	7.0 ± 0.3	6.9 ± 0.3	7.3 ± 0.5	7.0 ± 0.4
**SM**	35.7 ± 1.1	35.5 ± 1.1	36.5 ± 1.5	36.0 ± 1.4
**SMI**	12.6 ± 0.4	12.5 ± 0.4	13.3 ± 0.6	13.1 ± 0.5

**Table 3:** Hormonal variations in both groups at baseline and after 2 months of treatment. Data are reported as mean ± SEM. * *p* < 0.05 T2 vs. T0. Abbreviations: cholecystokinin (CCK).

	Plasma Hormonal Parameters			
	Treated Group (n = 32)		Placebo Group (n = 27)	
	T0	T2	T0	T2
**CCK pg/mL (± SEM)**	14.21 ± 3.4	13.26 ± 1.3	14.11 ± 2.21	5.66 ± 1.10 *
**Ghrelin ng/mL (± SEM)**	7.84 ± 2.6	7.63 ± 2.5	7.53 ± 1.21	7.66 ± 1.40

## Data Availability

The data are stored in a database at the Department of Clinical Medicine and Surgery, University of Naples “Federico II”, 80131 Naples, Italy. The data are available on request from the corresponding author.
